# Supplementary material for: Unconventional exciton evolution from the pseudogap to superconducting phases in cuprates
Source: Nat Commun. 2022 Dec 23;13:7906. doi: 10.1038/s41467-022-35210-8 (PMC9780265; doi:10.1038/s41467-022-35210-8)
Supplement: Supplementary file 1 — Supplementary Information [file 41467_2022_35210_MOESM1_ESM.pdf]

Supplementary Information for

Unconventional exciton evolution from the pseudogap to superconducting phases in cuprates

A. Singh, H. Y. Huang, J. D. Xie, J. Okamoto, C. T. Chen,
T. Watanabe, A. Fujimori[§], M. Imada[†], and D. J. Huang[*]

[§]email: fujimori@phys.s.u-tokyo.ac.jp
[†]email: imada@ap.t.u-tokyo.ac.jp
[*]email: djhuang@nsrrc.org.tw

November 16, 2022

This PDF file includes:

Electronic structure of cuprates

Materials and Methods

Figures S1 to S9

References (1-18)

Electronic structure of cuprates

Figure S1 shows spectral function and dispersion of a hole-doped square-lattice calcuated from the Hubbard model. The hopping integral and the on-site Coulomb interaction used to simulate the cuprates are $t = 0.5$ eV and $U = 4$ eV, respectively. Single-particle spectral function along a symmetric momentum line are reproduced from Fig. 7(a) of Ref. [1] at hole doping 12.5%. The calculations were performed by a variational Monte-Carlo method. The coherent LHB and the incoherent LHB are denoted by "coh LHB" and "incoh LHB," respectively. Figure S1**b** plots dispersions of the coherent LHB, the IGB, and a dark fermion band in the pseudogap energy region indicated by the blue dashed lines, adopted from Fig. 2(a) of Ref. [2], where the cluster dynamical mean-field method was employed for hole doping 9%. Occupied regions for an electron are highlighted in blue. The green surfaces plot the poles of the single-particle Green's function $G(\mathbf{k}, \omega)$, showing peaks of single-particle spectral weights. The red surface plots the zeros of $G(\mathbf{k}, \omega)$, which generates the pseudogap in this energy region and represents the dark fermion band in the present interpretation. The in-plane momenta $\mathbf{k}$ are given in units of $1/a$, in which a is the lattice parameter.

In the calculations shown in Fig. S1**b**, the pseudogap is characterized by the existence of the dark fermion band plotted as the red surface representing the zeros of the Green's function. For the physical picture of the zero of Green's function and the dark fermion dispersion to capture the essence of the pseudogap, readers are referred to Sec. **Two-component fermion model** and Ref. [3].

Supplementary Methods

Samples

Optimally-doped single crystals of $Bi_{2.1}Sr_{1.9}CaCu_2O_{8+\delta}$ (Bi2212) were grown in air using the traveling solvent floating zone method [4]. The crystals were then annealed under an oxygen partial pressure of 100 Pa at 600° C to realize optimal doping with $T_c = 89$ K. The hole doping level of $p = 0.16 \pm 0.005$ was obtained using Tallon's empirical relation [5]. Figure S2 shows that a weak pseudogap opens below ∼220 K according to the c-axis resistivity and a strong pseudogap open below ∼160 K according to the ab-plane resistivity [6]. Pb-doped single crystals of $Bi_{1.6}Pb_{0.4}Sr_2CaCu_2O_{8+\delta}$ were also prepared: Optimally-doped samples with $T_c = 93$ K and $p = 0.16 \pm 0.005$, and overdoped ones with $T_c = 65$ K and $p = 0.22 \pm 0.005$. For the overdoped samples, a weak pseudogap opens below ∼130 K according to the c-axis resistivity but the ab-plane resistivity shows no signature of a strong pseudogap [6].

Cu L-edge and O K-edge RIXS measurements

We conducted Cu L-edge and O K-edge RIXS measurements using the AGM-AGS spectrometer of beamline 41A at Taiwan Photon Source of National Synchrotron Radiation Research Center, Taiwan. This recently constructed AGM-AGS beamline is based on the energy compensation principle of grating dispersion; it has achieved an energy resolution of 16 meV at 530 eV photon energy [7]. The instrument energy resolution was 90 meV FWHM for Cu L-edge RIXS measurements.

We used RIXS to measure the excitonic excitation of Bi2212. Figure S3(a) shows a simplified graphic illustration of the excitation of a low-energy exciton in a hole-doped system induced by RIXS. The energy levels of all three stages of the RIXS excitation are aligned to E_{F}. A Cu $2p$ electron is excited to the IGB with an energy ω_0 above E_{F}, and then another electron in the coherent LHB decays to fill the $2p$ core hole through emitting another photon of energy ω'. The

UHB and incoherent LHB are pulled down by the $2p$ core hole in the intermediate state.

XAS spectra, as plotted in Fig. S3(b), were recorded at normal incidence with σ polarization by using the total electron yield method. RIXS spectra above and below T_c were recorded with σ polarized incident X-rays of which the polarization was perpendicular to the scattering plane.Prior to RIXS measurements, a clean sample surface (001) was obtained on cleaving the sample in air. For the RIXS measurements, the scattering angle was fixed to 150°. The a-axis and c-axis lay in the horizontal scattering plane while the b-axis was perpendicular to the scattering plane. The scattered X-rays were detected without a polarization analysis.

We extrapolated the fluorescence to a threshold, i.e., a zero energy loss, from the linear extrapolation of energy loss E_{loss} vs. incident photon energy. The fluorescence threshold lead us to identify the energy position corresponding to the center of the occupied Cu $3d$ band; we found that it is 2 eV below the XAS absorption peak L_3. Because LDA calculations [8] and resonant photoemission results [9] indicate that the center of the occupied Cu $3d$ band is $\sim$3 eV below the Fermi level E_{F}, the position of E_{F} in the absence of the core-hole potential is determined to be 1 eV above L_3. Our observation is consistent with the conclusions of the Cu $2p$ core-level photoemission [10] and absorption peak energies [11], which suggest that the Cu L_3 peak is located $\sim$1 eV below E_{F}. Therefore, if we assume that relevant electronic states near E_{F} such as states in the IGB and coh LHB are shifted downwards by $\sim$1 eV owing to the core-hole potential, the energies of these electronic states relative to E_{F} are given by the energies relative to the L_3 absorption peak, as illustrated in Fig. S3(c).

Supplementary XAS and RIXS data are presented in Figs. S4, S5, and S6, for O K-edge XAS spectrum of Bi-2212, Cu L_3-edge RIXS of optimally doped Bi2212 with $\mathbf{Q}_{\|} = (\pi/2, 0)$ and over-doped Pb-Bi2212 with $\mathbf{Q}_{\|} = (\pi, 0)$, respectively.

To analyze the detailed temperature dependence of RIXS spectra across the superconducting transition, we carried out an exciton spectral weight analysis using different energy win-

dows. The spectral weight of exciton is defined as the integration of RIXS spectra for a selected region in energy loss. To estimate the error, we integrated RIXS spectra with different energy loss windows, as shown in Fig. S8. The exciton spectral weights normalized to one for 25 and and 250 K are, respectively, plotted in Figs. S8a and S8b. The error bar of each temperature is taken as the average of the variations in exciton spectral weights of both normalization schemes deduced from those selected energy loss regions.

Two-component fermion model

For the self-contained description, we summarize the structure of the Green's function and the self-energy of the two-component fermion model (TCFM) by referring to Refs. [12, 13, 3]. The TCFM in the normal state is defined by the following Hamiltonian:

$$\begin{aligned} H &= \sum_{k,\sigma,\sigma'} [\epsilon_c(k) c^\dagger_{k,\sigma} c_{k,\sigma} + \Lambda(k)(c^\dagger_{k,\sigma} d_{k,\sigma} + \text{H.c.}) \\ &+ \epsilon_d(k) d^\dagger_{k,\sigma} d_{k,\sigma}]. \end{aligned} \tag{S1}$$

Here, the fermion c represents the original quasparticle with the dispersion $\epsilon_c(k)$ at the momentum k in a form of a noninteracting Hamiltonian. The dark fermion represented by d with the dispersion $\epsilon_d(k)$ emerging from the strong correlation of the electrons hybridizes to the fermion c via the coupling $\Lambda(k)$.

In the present interpretation of the doped cuprates, the original single-band system representing the antibonding band generated by strongly hybridized Cu $3d_{x^2-y^2}$ and O $2p_\sigma$ orbitals is under strong correlation. Then the interaction effect primarily yields emergent electron fractionalization, which results in the splintered two fermion degrees of freedom, c and d in the low-energy degrees of freedom near the fermi level. The fermion c, the normal quasiparticle component is built from the bare electron of antibonding band, while the strong correlation near the Mott insulator generates the effect beyond the conventional quasiparticle picture, which is

better represented by the electron fractionalization. The emergent d fermion represents such effects. The split of the degrees of freedom was, as one possibility, proposed to be generated from the electron bistability in the underdoped region. The bistability may also be the origin of the charge inhomogeneity or charge order widely observed in the cuprates [14]. The fractionalization effect is represented in the original quasiparticle c by the self-energy Σ, which we derive below.

Although Eq.(S1) can be an effective phenomenological Hamiltonian of the symmetry broken phases such as stripe, nematic or time reversal symmetry broken states in the mean-field approximation, we should note that c and d are both visible particles by the spectroscopic measurements such as ARPES and the present RIXS in this case. This is because, for instance, d_k is simply c_{k+Q} in the translational symmetry broken phase with the ordering wave vector Q. In fact, such a symmetry broken phase does not yield the enhancement of the RIXS intensity we observed because the summation of detectable c and d density of states is preserved. The enhancement is purely a consequence of indetectabe nature of the d fermion by the conventional spectroscopic measurements. The electron fractionalization, we assume, is then a consequence of NOT the symmetry breaking but simply the Mottness effect without the symmetry breaking. The degrees of freedom d is related to the broad background-like incoherent component in the spectroscopic measurements. The electron fractionalization gets increasing support as we summarize below.

The single-particle dynamics of the original quasiparticle c in Eq.(S1) is represented by the Green's function in the form of

$$G_c(k,\omega) = \frac{1}{\omega - \epsilon(k) - \Sigma(k,\omega)}, \tag{S2}$$

with the self-energy

$$\Sigma(k,\omega) = \frac{\Lambda(k)^2}{\omega - \epsilon_d(k)}. \tag{S3}$$

Equation (S3) indicates that the pole of the self-energy emerges at $\omega = \epsilon_d(k)$, namely at the bare dispersion of the fermion d. This pole generates the zero of G and a gap in the density of states of the fermion c known as the hybridization gap, which is given as

$$\Delta_{\text{HG}} = \sqrt{(\epsilon_c(k) - \epsilon_d(k))^2 + 4\Lambda(k)^2}. \tag{S4}$$

In the TCFM picture, the pseudogap observed in the cuprates is well understood by this hybridization gap as is illustrated in Fig. S9(b). Here the original visible quasiparticle c is detected as the two split bands with the dispersion

$$\epsilon_{\text{IGB}} = \frac{1}{2}(\epsilon_c + \epsilon_d + \sqrt{(\epsilon_c - \epsilon_d)^2 + 4\Lambda^2} \tag{S5}$$

$$\epsilon_{\text{cohLHB}} = \frac{1}{2}(\epsilon_c + \epsilon_d - \sqrt{(\epsilon_c - \epsilon_d)^2 + 4\Lambda^2}, \tag{S6}$$

where ϵ_{IGB} and ϵ_{cohLHB} represents the ingap band above the fermi level and the coherent LHB crossing the fermi level, respectively.

To gain insight into the nature of the pseudogap formation, it is useful to examine the superconducting phase as well by introducing the d-wave superconducting mean field as

$$\begin{aligned} H &= \sum_{k,\sigma}[\epsilon_c(k)c^\dagger_{k,\sigma}c_{k,\sigma} + \epsilon_d(k)d^\dagger_{k,\sigma}d_{k,\sigma} \\ &+ \Lambda(k)(c^\dagger_{k,\sigma}d_{k,\sigma} + \text{H.c.}) \\ &+ (\Delta_c(k)c^\dagger_{k,\sigma}c^\dagger_{-k,-\sigma} + \Delta_d(k)d^\dagger_{k,\sigma}d^\dagger_{-k,-\sigma} + \text{H.c})], \end{aligned} \tag{S7}$$

where the anomalous part proportional to the superconducting order parameters $\Delta_c(k)$ and $\Delta_d(k)$ becomes nonzero. This is nothing but Eq.(1) in the main text.

Then Green's function for c particle in Nambu representation is obtained as

$$G_c(k,\omega) = \frac{1}{\omega - \epsilon_c(k) - \Sigma^{\text{nor}}(k,\omega) - W(k,\omega)}, \tag{S8}$$

with

$$W(k,\omega) = \frac{\Sigma^{\rm ano}(k,\omega)^2}{\omega + \epsilon_c(k) + \Sigma^{\rm nor}(k,-\omega)^*}, \tag{S9}$$

$$\Sigma^{\rm nor}(k,\omega) = \frac{\Lambda(k)^2(\omega + \epsilon_d(k))}{\omega^2 - \epsilon_d(k)^2 - \Delta_d(k)^2}, \tag{S10}$$

and

$$\Sigma^{\rm ano}(k,\omega) = \Delta_c(k) - \frac{\Lambda(k)^2 \Delta_d(k)}{\omega^2 - \epsilon_d(k)^2 - \Delta_d(k)^2}. \tag{S11}$$

Now the pole position of $\Sigma^{\rm nor}$ at $\omega = \epsilon_d(k)$ in the normal state (expected to generate the pseudogap) is modified to $\omega = \pm\sqrt{\epsilon_d(k)^2 + \Delta_d(k)^2}$. Remarkably, the anomalous part $\Sigma^{\rm ano}$ also has a pole exactly at the same position. Accordingly, W (Eq.(S9)) in the denominator of G in Eq.(S8) also has a pole of the order 1 at the same energy. The residue of the poles of W and $\Sigma^{\rm nor}$ at $\omega = \pm\sqrt{\epsilon_d(k)^2 + \Delta_d(k)^2}$ are given by $\frac{\Lambda^2}{2}\left(1 \pm \frac{\epsilon_d}{\sqrt{\epsilon_d^2+\Delta_d^2}}\right)$ and $-\frac{\Lambda^2}{2}\left(1 \pm \frac{\epsilon_d}{\sqrt{\epsilon_d^2+\Delta_d^2}}\right)$, respectively. Therefore, their residues cancel out in their sum in Eq. (S8) and will be hidden in the direct data of the spectral function measured by ARPES [13, 3]. If the origin of the pseudogap is not ascribed to this fractionalization mechanism, such a remarkable cancellation would not be expected. The hidden pole structure was extracted by machine learning of ARPES data in Ref. [15].

In the present analysis, as are described in Eqs.(2) and (3) in the main text, we have employed the parameters of the TCFM [16] as follows:

$$\begin{aligned}
\epsilon_c(k) &= -(2t_{c1}(\cos k_x + \cos k_y) + 4t_{c2}\cos k_x \cos k_y) + \mu_c, \\
\epsilon_d(k) &= -(2t_{d1}(\cos k_x + \cos k_y) + 4t_{d2}\cos k_x \cos k_y) + \mu_d, \\
\Lambda(k) &= \Lambda_0 + \Lambda_1(\cos k_x + 1)(\cos k_y + 1),
\end{aligned} \tag{S12}$$

$$\begin{aligned}
\Delta_c(k) &= \frac{\Delta_{c0}}{2}(\cos k_x - \cos k_y), \\
\Delta_d(k) &= \frac{\Delta_{d0}}{2}(\cos k_x - \cos k_y),
\end{aligned} \tag{S13}$$

where the parameters are chosen to reproduce the ARPES data[17] analyzed by machine learning [15] and STM data [18] as $t_{c1} = 0.1953$, $t_{c2} = -0.0762$, $t_{d1} = 0.0100$, $t_{d2} = -0.0036$, $\mu_c = 0.2175$, $\mu_d = 0.0105$, $\Delta_{c0} = 0.02$, $\Delta_{d0} = 0.07$, $\Lambda_0 = 0.0658$ and $\Lambda_1 = -0.014$ in the unit of eV. In fact, these parameters well fit the ARPES data [17] and the machine learning analysis [15]. For instance the well-known peak-dip hump structure of the spectral weight $\mathrm{Im}G(k,\omega)$ for Bi2212 in the superconducting phase is quantitatively reproduced as is shown in Fig. S9. Here the quasiparticle peak energy is $\pm \sim 0.03$ eV and the hump energy at the antinodal point is around ± 0.12 ev in agreement with the ARPES data [17]. Furthermore, the particle-hole asymmetric structure of the density of states observed in scanning tunneling microscope [18] as well as the pseudogap size above $T_{\rm c}$ larger than the superconducting gap below $T_{\rm c}$ is also reproduced.

For the cancellation of the normal and anomalous component of the self-energy in the spectral function as is indeed the case of the machine learning result of the ARPES measurement, we are forced to employ the electron fractionalization. However, the ARPES data contains several limitations such as the range limit of detectable momentum and energy windows, uncertainty arising from unknown background effects and experimental noise. Therefore, it is desired to perform independent measurement by RIXS to stringently test the radical concept of the fractionalization, because of its fundamental importance.

Supplementary References

[1] Charlebois, M. & Imada, M. Single-Particle Spectral Function Formulated and Calculated by Variational Monte Carlo Method with Application to d-Wave Superconducting State. *Phys. Rev. X* **10**, 041023 (2020).

[2] Sakai, S., Motome, Y. & Imada, M. Evolution of Electronic Structure of Doped Mott

Insulators: Reconstruction of Poles and Zeros of Green's Function. *Phys. Rev. Lett.* **102**, 056404 (2009).

[3] Imada, M. & Suzuki, T. J. Excitons and Dark Fermions as Origins of Mott Gap, Pseudogap and Superconductivity in Cuprate Superconductors—General Concept and Basic Formalism Based on Gap Physics. *J. Phys. Soc. Jpn.* **88**, 024701 (2019).

[4] Watanabe, T., Fujii, T. & Matsuda, A. Anisotropic resistivities of precisely oxygen controlled single-crystal $Bi_2Sr_2CaCu_2O_{8+\delta}$: Systematic study on"spin gap"effect. *Phys. Rev. Lett.* **79**, 2113 (1997).

[5] Obertelli, S., Cooper, J. & Tallon, J. Systematics in the thermoelectric power of high-T_c oxides. *Phys. Rev. B* **46**, 14928 (1992).

[6] Usui, T. *et al.* Doping dependencies of onset temperatures for the pseudogap and superconductive fluctuation in $Bi_2Sr_2CaCu_2O_{8+\delta}$, studied from both in-plane and out-of-plane magnetoresistance measurements. *J. Phys. Soc. Jpn.* **83**, 064713 (2014).

[7] Singh, A. *et al.* Development of the soft X-ray AGM-AGS RIXS beamline at the taiwan photon source. *J. Synchrotron Radiat.* **28**, 977 (2021).

[8] Krakauer, H. & Pickett, W. E. Effect of bismuth on high-T_c cuprate superconductors: Electronic structure of $Bi_2Sr_2CaCu_2O_8$. *Phys. Rev. Lett.* **60**, 1665–1667 (1988).

[9] Tjeng, L. H., Chen, C. T. & Cheong, S.-W. Comparative soft-x-ray resonant-photoemission study on $Bi_2Sr_2CaCu_2O_8$, CuO, and CuO_2. *Phys. Rev. B* **45**, 8205–8208 (1992).

[10] Sekhar, B. *et al.* Core-level X-ray photoemission studies of $Bi_2Sr_2Ca_{1-x}Eu_xCu_2O_y$. *Physica C* **206**, 139–147 (1993).

[11] N. Nücker, N. *et al.* Symmetry of holes in high-T_c superconductors. *Phys. Rev. B* **39**, 6619–6629 (1989).

[12] Yang, K.-Y., Rice, T. M. & Zhang, F.-C. Phenomenological theory of the pseudogap state. *Phys. Rev. B* **73**, 174501 (2006).

[13] Sakai, S., Civelli, M. & Imada, M. Hidden Fermionic Excitation Boosting High-Temperature Superconductivity in Cuprates. *Phys. Rev. Lett.* **116**, 057003 (2016).

[14] Imada, M. Charge Order and Superconductivity as Competing Brothers in Cuprate High-T_c Superconductors. *J. Phys. Soc. Jpn.* **90**, 111009 (2021).

[15] Yamaji, Y., Yoshida, T., Fujimori, A. & Imada, M. Hidden self-energies as origin of cuprate superconductivity revealed by machine learning. *Phys. Rev. Research* **3**, 043099 (2021).

[16] Imada, M. Resonant Inelastic X-Ray Scattering Spectra of Cuprate Superconductors Predicted by Model of Fractionalized Fermions. *J. Phys. Soc. Jpn.* **90**, 074702 (2021).

[17] Kondo, T. *et al.* Disentangling Cooper-pair formation above the transition temperature from the pseudogap state in the cuprates. *Nature Physics* **7**, 21 (2011).

[18] Fischer, O., Kugler, M., Maggio-Aprile, I., Berthod, C. & Renner, C. Scanning tunneling spectroscopy of high-temperature superconductors. *Rev. Mod. Phys.* **79**, 353 (2007).

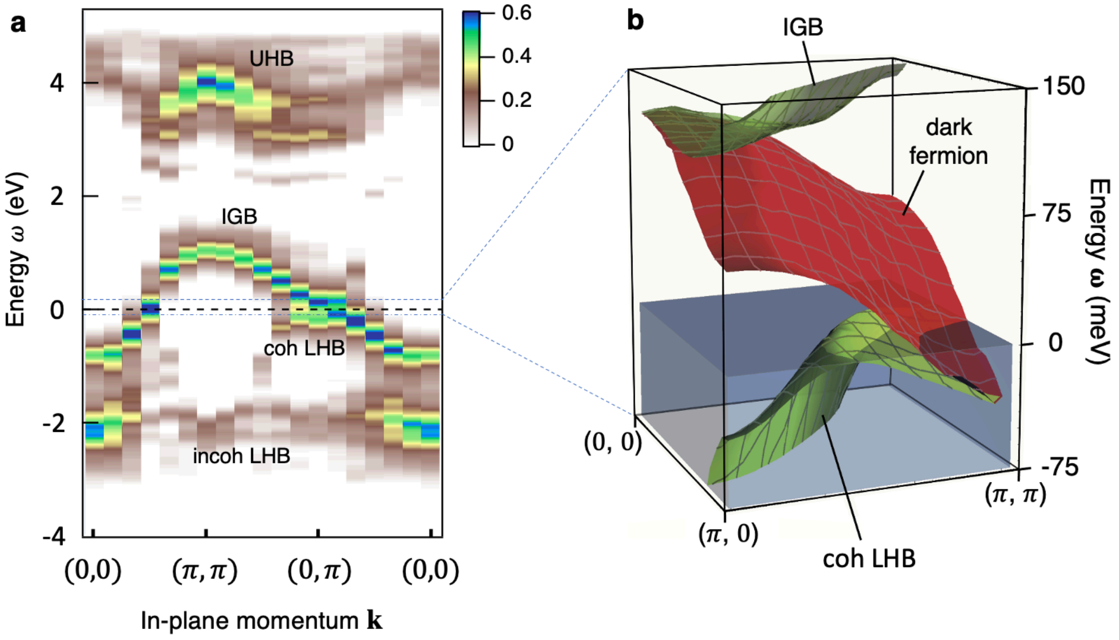

Figure S1: **a** Spectral function and dispersion of a hole-doped square-lattice Hubbard model. **b** Dispersions of the coherent LHB, the IGB, and a dark fermion band in the pseudogap energy region.

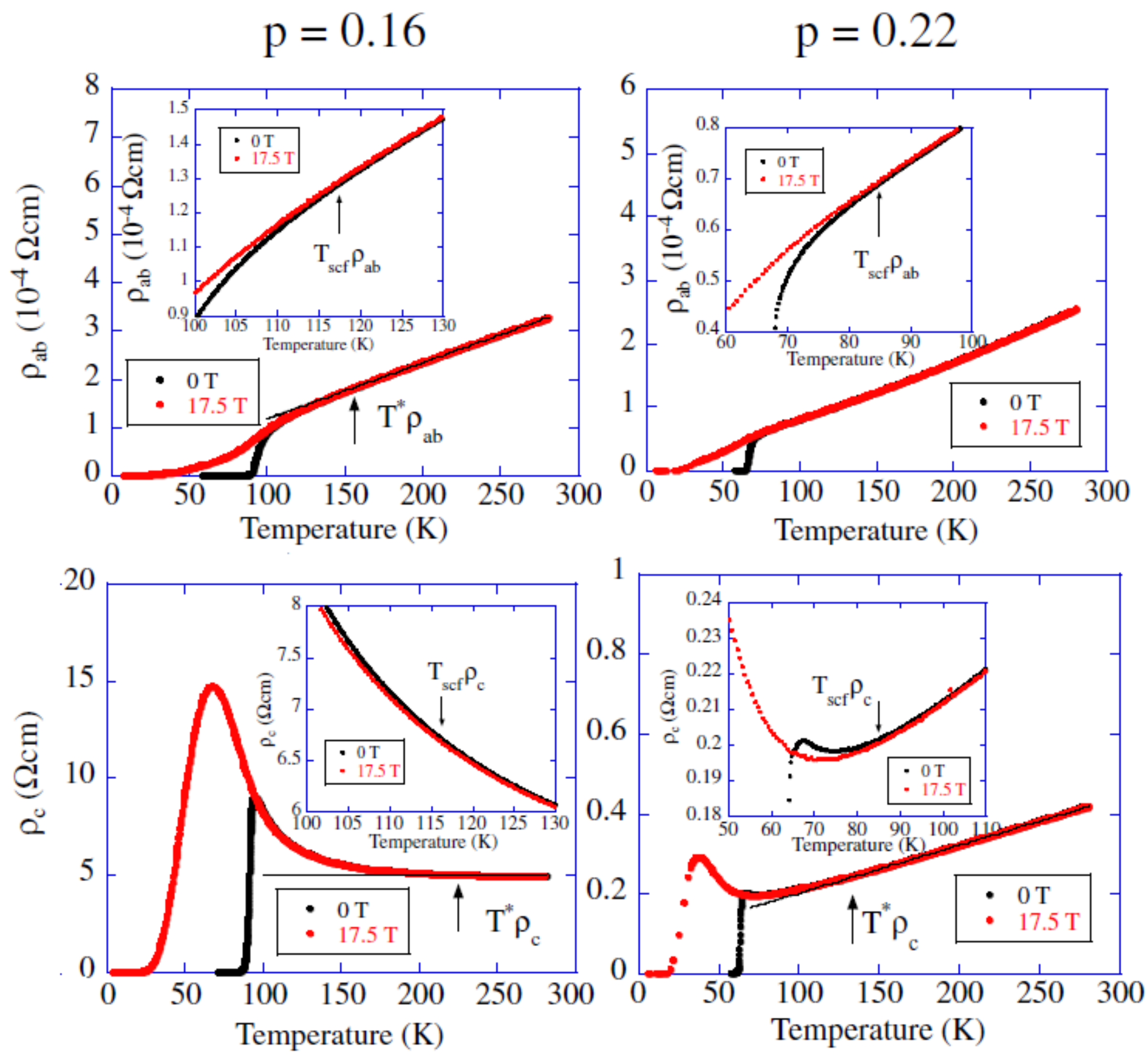

Figure S2: In-plane and out-of-plane resistivities ρ_{ab} and ρ_c of Bi2212 single crystals [6]. The temperatures $T^*_{\rho_{ab}}$ and $T^*_{\rho_c}$ at which the resistivity starts to deviate from the higher-temperature linear behaviors indicates the opening of pseudogaps. Left: Optimally-doped $Bi_{2.1}Sr_{1.9}CaCu_2O_{8+\delta}$ with $p = 0.16$. Right: Overdoped $Bi_{1.6}Pb_{0.4}Sr_2CaCu_2O_{8+\delta}$ with $p = 0.22$. No signature of the pseudogap opening is seen in the ρ_{ab} of the overdoped sample. In the inset, arrows indicate the temperatures $T_{\mathrm{scf}\rho_{\mathrm{ab}}}$ and $T_{\mathrm{scf}\rho_{\mathrm{c}}}$ below which superconducting fluctuations are significant and are suppressed by the magnetic field of 17.5 T.

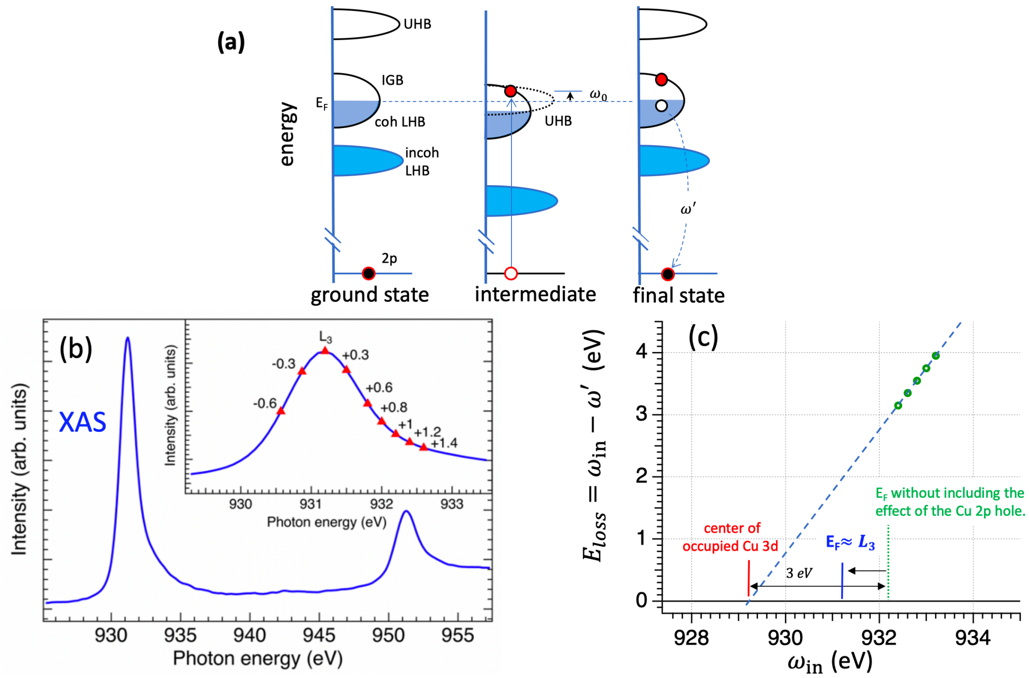

Figure S3: (a) Simplified graphic illustration of the excitation of a low-energy exciton in a hole-doped system induced by RIXS. The energy levels of all three stages of the RIXS excitation are aligned to E_F. (b) The Cu L-edge XAS spectrum of Bi-2212 measured with σ polarization by using the total electron yield method. Inset: zoom-in XAS spectrum around L_3-edge; the peak energy is denoted as L_3. Red triangles indicate the energies used for the RIXS measurements. (c) Estimate the energy position of E_F in the Cu L_3-edge absorption spectrum through the fluorescence threshold from the linear extrapolation of energy loss E_loss vs incident photon energy ω_in.

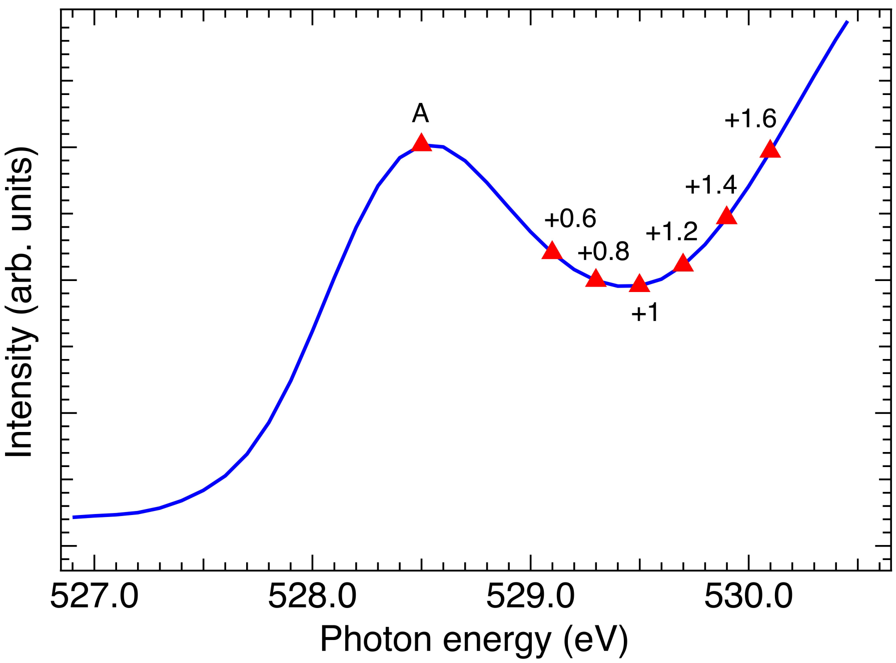

Figure S4: The O K-edge XAS spectrum of Bi-2212 measured with σ polarization by using the total electron yield method. The resonant energy is denoted as A. Red triangles indicate the energies used for the RIXS measurements.

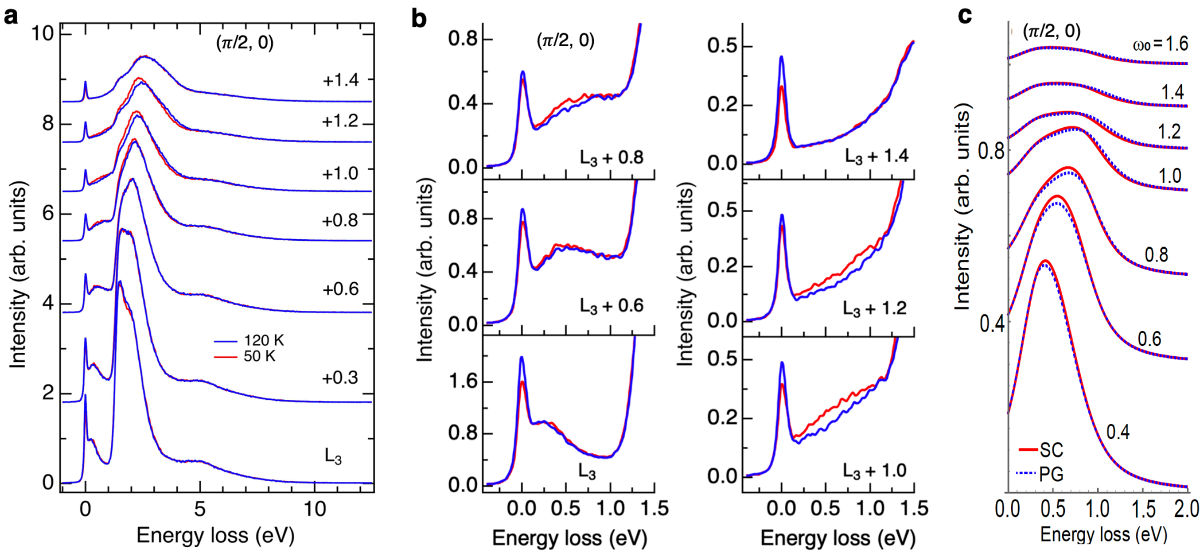

Figure S5: **Enhancement of excitonic excitations in Cu L_3-edge RIXS of optimally doped Bi2212 by superconductivity**. **a** & **b** RIXS spectra at selected incident photon energies for $\mathbf{Q}_\parallel = (\pi/2, 0)$ and temperatures above and below T_c = 89 K. The incident photon energy is denoted as its energy above the L_3 absorption in units of eV. Spectra above and below T_c are normalized for energy loss from 1.7 eV to 13 eV. From the estimate shown in supplementary Fig. S2, L_3 corresponds approximately to E_F. **c** Calculated RIXS resulting from excitonic excitations in the superconducting (SC) and pseudogap (PG) phases for $\mathbf{Q}_\parallel = (\pi/2, 0)$. The incident energy ω_0 measured from E_F is given in units of eV. The core-hole lifetime width Γ and the broadening factor η were set to 0.3 eV and 0.1 eV, respectively. All spectra are plotted with a vertical offset for clarity.

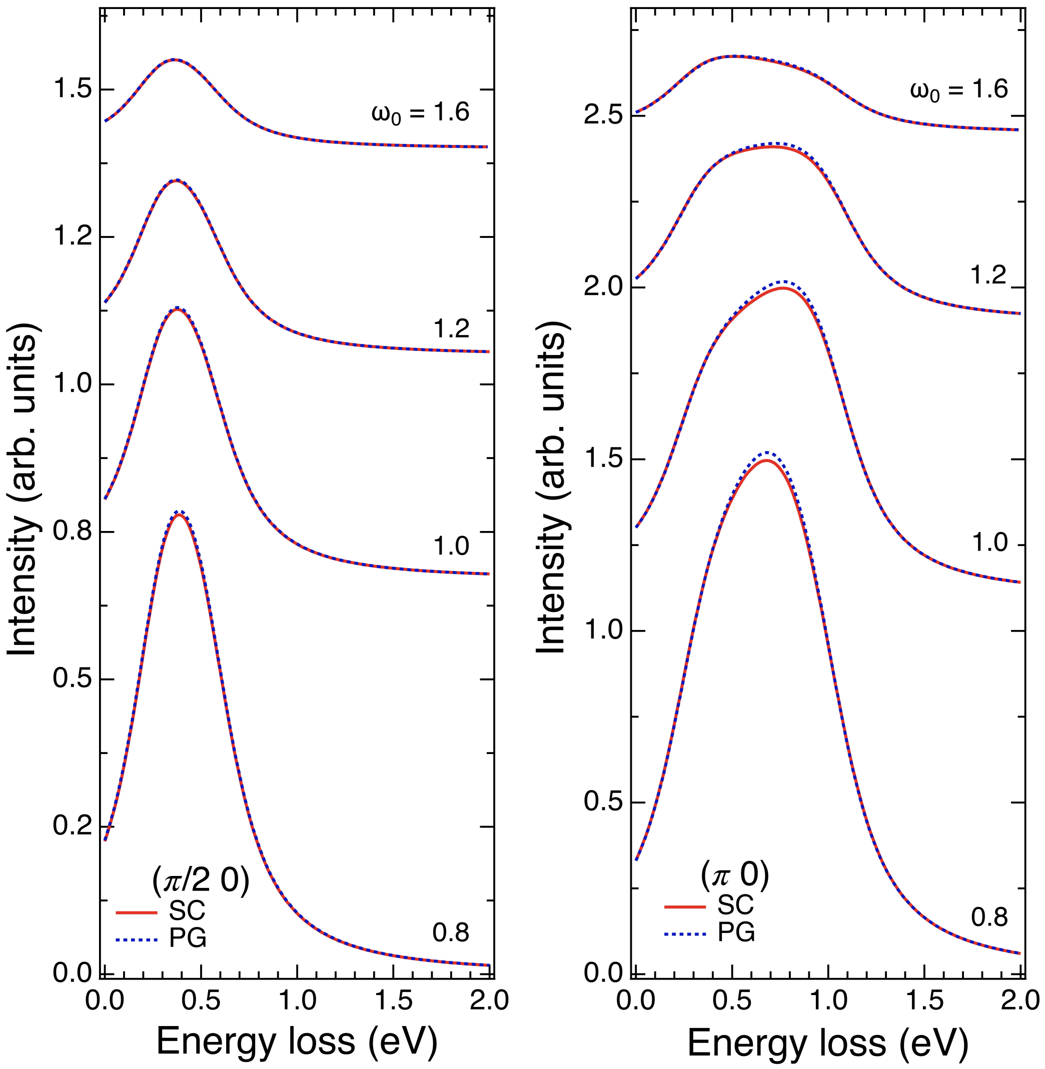

Figure S6: Calculated RIXS spectra using a conventional single-component Hubbard model for SC and PG phases at selected ω_0 for $\mathbf{Q}_\parallel = (\pi/2, 0)$ and $(\pi, 0)$. All spectra are plotted with a vertical offset for clarity.

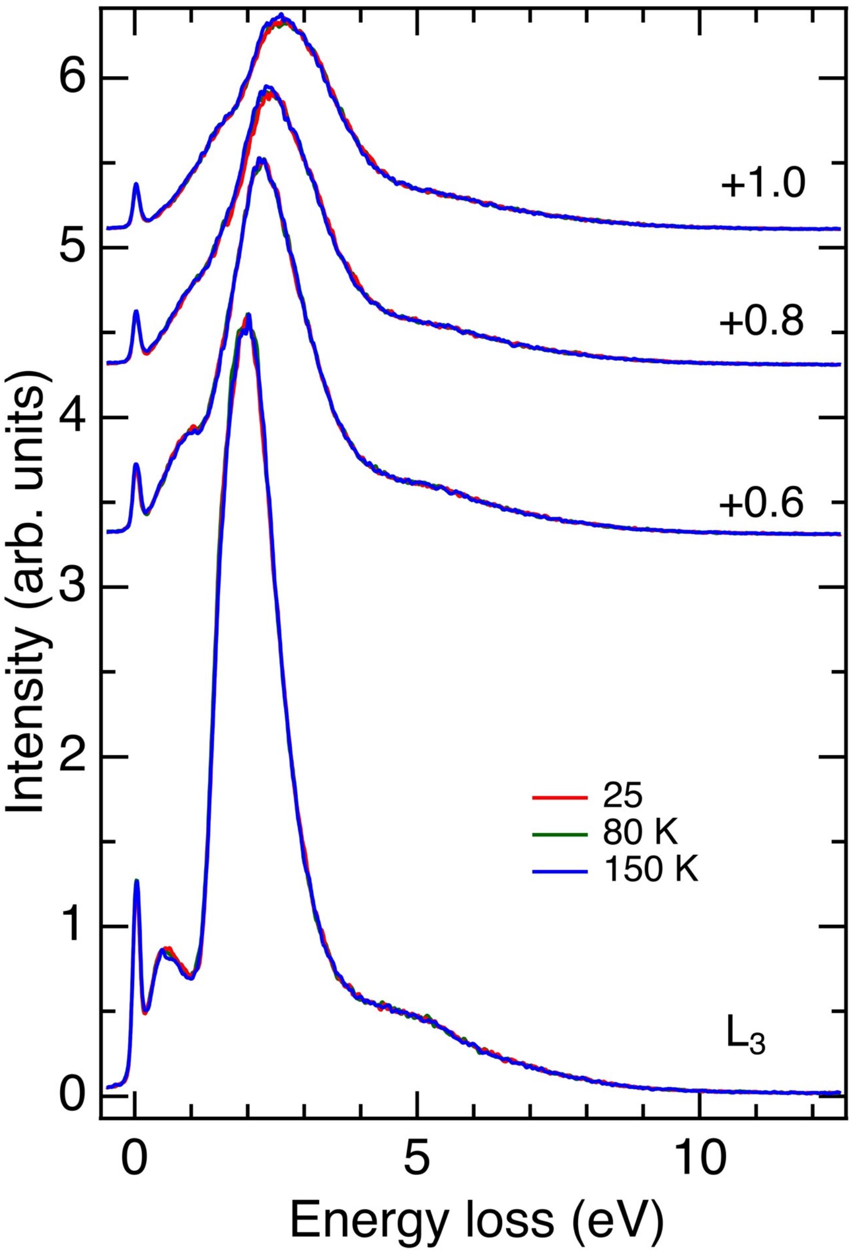

Figure S7: Cu L_3-edge RIXS spectra of OD Pb-Bi2212 measured at $\mathbf{Q}_{\parallel} = (\pi, 0)$ for various incident energies across the L_3 peak of the XAS. RIXS spectra were measured at 25K, 80K and 150K as plotted by red green and blue solid lines. Spectra are plotted with a vertical offset for clarity.

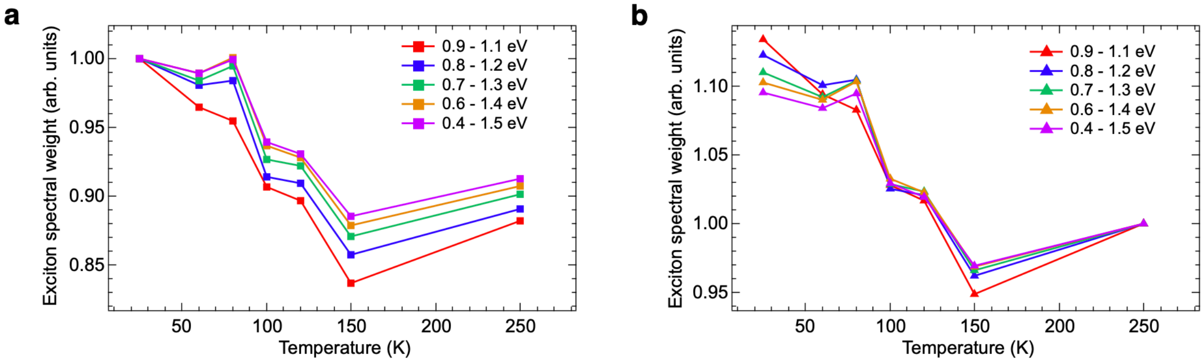

Figure S8: Evolution of the exciton spectral weight deduced from different energy-loss windows for the OP sample. **a** The exciton spectral weight normalized to one for 25 K. **b** The exciton spectral weight normalized to one for 250 K.

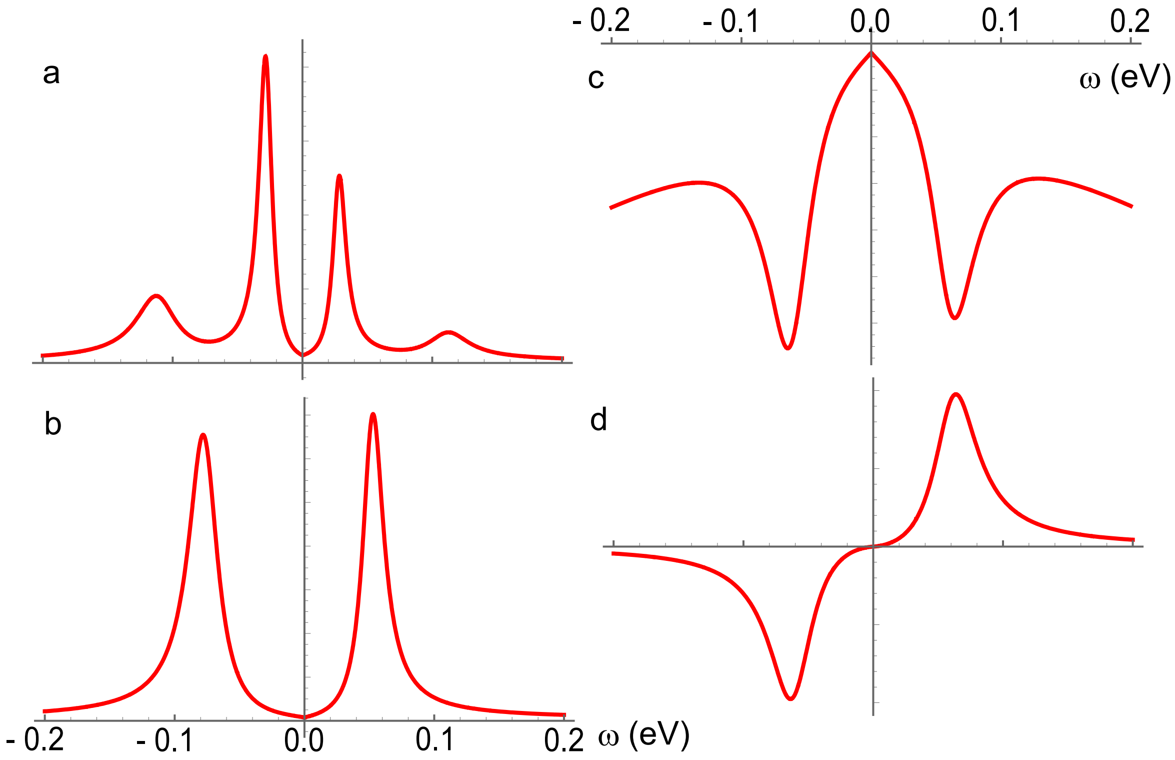

Figure S9: **a** Spectral function $A(k,\omega)$ obtained from TCFM in the superconducting phase to reproduce the ARPES [17] and the machine learning data [15]. **b** $A(k,\omega)$ in the normal (pseudogap) state for the same parameters as **a** except for the choice $\Delta_{d0} = \Delta_{c0} = 0$. **c**. Imaginary part of the normal self-energy for the superconducting state. **d** Imaginary part of the anomalous self-energy for the superconducting state. In the superconducting state normal and anomalous contributions of the peaks to $A(k,\omega)$ cancels.
